# Bispuupehenone from the South Chinese Sea sponge *Dysidea* sp.

**DOI:** 10.1107/S1600536808011987

**Published:** 2008-04-30

**Authors:** Song Qin, Lei Shi, Jia Li, Yue-Wei Guo

**Affiliations:** a State Key Laboratory of Drug Research, Shanghai Institute of Materia Medica, Chinese Academy of Sciences, Shanghai 201203, People’s Republic of China; b Chinese National Center for Drug Screening, Shanghai Institute of Materia Medica, Chinese Academy of Sciences, Shanghai 201203, People’s Republic of China

## Abstract

Bispuupehenone, C_42_H_54_O_6_, formally results from dimerization of puupehenone, which is constructed of sesquiterpene and benzene units. Bispuupehenone was isolated from the South China Sea sponge *Dysidea* sp. and the single-crystal X-ray diffraction analysis confirmed the previously reported structure. The mol­ecule is located on a twofold axis and the dimerization forms two fused dibenzopyran systems related by symmetry. In the asymmetric unit, the two cyclohexane rings adopt chair conformations, while the two pyran rings adopt half-chair conformations. The relative stereochemistry and configurations for the ring junctions are in agreement with the structure reported previously.

## Related literature

The title compound was first isolated from the Pacific marine sponge *Heteronema* sp., see Amade *et al.* (1983 ▶). For the biological and pharmaceutical activity of puupehenone, see: Barrero *et al.* (1998 ▶, 1999 ▶); Castro *et al.* (2004 ▶); Ciavatta *et al.* (2007 ▶); Longley *et al.* (1993 ▶); Kohmoto *et al.* (1987 ▶); Takamatsu *et al.* (2003 ▶). For the synthesis and semi-synthesis of puupehenone and its derivatives, see: Hamann (2003 ▶); Alvarez-Manzaneda *et al.* (2005 ▶, 2007 ▶).
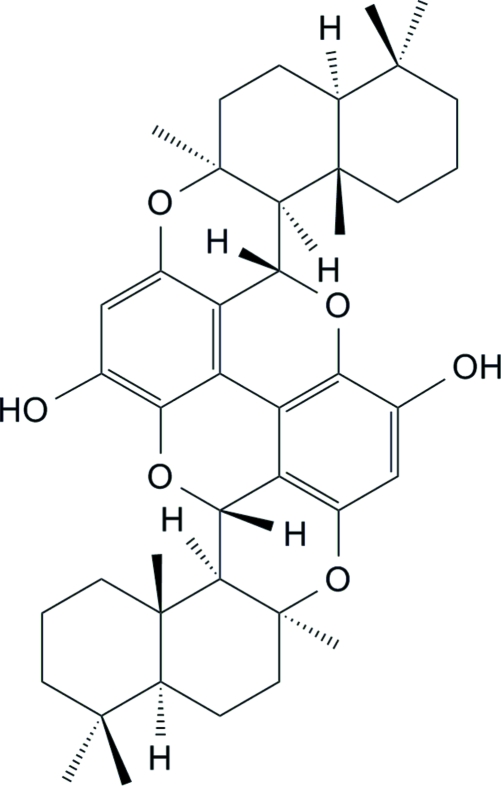


## Experimental

### 

#### Crystal data

C_42_H_54_O_6_
*M* *_r_* = 654.85
Tetragonal, 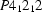

*a* = 13.5981 (10) Å
*c* = 18.7260 (19) Å
*V* = 3462.6 (5) Å^3^
*Z* = 4
Mo *K*α radiation
μ = 0.08 mm^−1^
*T* = 293 (2) K
0.39 × 0.24 × 0.14 mm

#### Data collection

Bruker APEX CCD area-detector diffractometer
Absorption correction: multi-scan (*SADABS*; Bruker, 2000 ▶) *T*_min_ = 0.743, *T*_max_ = 0.990
20532 measured reflections
2219 independent reflections
1644 reflections with *I* > 2σ(*I*)
*R*_int_ = 0.110

#### Refinement

*R*[*F* ^2^ > 2σ(*F* ^2^)] = 0.045
*wR*(*F* ^2^) = 0.113
*S* = 0.94
2219 reflections
225 parameters
H atoms treated by a mixture of independent and constrained refinement
Δρ_max_ = 0.23 e Å^−3^
Δρ_min_ = −0.20 e Å^−3^

### 

Data collection: *SMART* (Bruker, 2000 ▶); cell refinement: *SAINT* (Bruker, 2000 ▶); data reduction: *SAINT*; program(s) used to solve structure: *SHELXS97* (Sheldrick, 2008 ▶); program(s) used to refine structure: *SHELXL97* (Sheldrick, 2008 ▶); molecular graphics: *SHELXTL* (Sheldrick, 2008 ▶); software used to prepare material for publication: *SHELXTL*.

## Supplementary Material

Crystal structure: contains datablocks I, global. DOI: 10.1107/S1600536808011987/bh2164sup1.cif

Structure factors: contains datablocks I. DOI: 10.1107/S1600536808011987/bh2164Isup2.hkl

Additional supplementary materials:  crystallographic information; 3D view; checkCIF report

## Figures and Tables

**Fig. 1.**
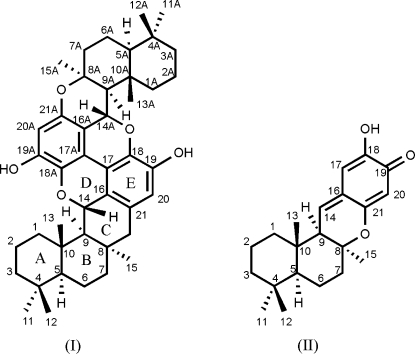
The structures of bispuupehenone (I) and puupehenone (II).

**Fig. 2.**
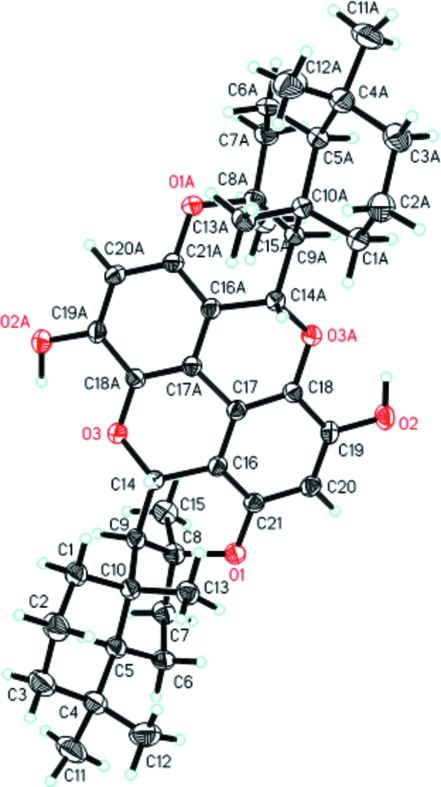
The projection of (I) showing the atom-labeling scheme.

## supplementary crystallographic information

### Comment

Bispuupehenone (I) was firstly isolated from Pacific marine sponge *Heteronema sp*. (Amade *et al.*, 1983), and was considered to be generated from co-occurring puupehenone (II) (Fig. 1) by *in vitro* oxidative coupling. The benzopyrane structure for the dimer is deduced by the comparison of its UV spectrum data with those of a simple dibenzofuran and a simple benzopyran. Although compound (II) and its derivatives have been reported to display a wide range of important biological activities, including antiviral, antifungal, antimalarial, and antitumor activities (Barrero *et al.*, 1998, 1999; Longley *et al.*, 1993; Castro *et al.*, 2004; Ciavatta *et al.*, 2007; Kohmoto *et al.*, 1987; Takamatsu *et al.*, 2003), the biological properties of (I) have been seldom reported. Synthesis and semi-synthesis of puupehenone and its derivatives have been published (Hamann, 2003); Alvarez-Manzaneda *et al.*, 2005, 2007).

As part of our research project on the study of the South China Sea marine organisms, a sample of the sponge *Dysidea sp*. was collected off the Lingshui Bay, Hainan Province, China, and was chemically investigated. Bispuupehenone, (I), was isolated and crystallized from the Et_2_O-soluble fraction of the acetone extract of the animal, and the structure of (I) was firstly elucidated by spectroscopic methods, NMR, UV and MS, and eventually confirmed through X-ray diffraction analysis. Herein, we report the X-ray structure of (I).

The projection of bispuupehenone is shown in Figure 2. In the structure, two puupehenone moieties are connected through two O atoms and a C—C bond between benzene rings, forming a benzopyran moiety at the midpoint of the axial symmetric molecule. Rings A and B of (I) are in chair conformations, while rings *C* and *D* adopt half-chair conformations. Moreover, the *trans* junction between rings *A*/*B* and the *cis* junction between rings *B*/*C* are in agreement with the structure reported previously.

Bispuupehenone was tested for the inhibitory activities against hPTP1B (human protein tyrosine phosphatase 1B), a key target for the treatment of Type-II diabetes and obesity, and showed excellent inhibitory effect with IC_50_ value of 0.98 mg ml^-1^. Other bioassays, such as antibacterial and anti-inflammatory, are currently ongoing.

### Experimental

The specimens of sponge were collected from Lingshui Bay, Hainan Province, China, in July 2004, and identified as *Dysidea sp*. by Professor J.-H. Li of the Institute of Oceanology, Chinese Academy of Sciences. A voucher specimen (LS-210) is available for inspection at the Herbarium of Shanghai Institute of Materia Medica, CAS. The frozen animals (dry weight 96.3 g) were cut into small pieces and exhaustively extracted with acetone (3×3 *L*). The organic extract was evaporated to give a residue, which was partitioned between Et_2_O and H_2_O. The Et_2_O solution was concentrated under reduced pressure to give a dark brown residue (4.7 g), which was fractionated by gradient silica gel column chromatography [0–100% acetone in light petroleum ether (PE)], yielding seven fractions (A···G). The fraction C eluted by PE/Me_2_CO (95:5) was further purified on a second silica gel column chromatography eluting with PE—Et_2_O (90:10) to afford (I) (14.3 mg). Crystals suitable for X-ray analysis were obtained by slow evaporation from a chloroform solution.

### Refinement

The non-H atoms were located in successive difference Fourier syntheses. The final refinements were performed by full-matrix least-squares methods with isotropic thermal parameters for all non-H atoms. Hydroxyl H atom H2 was found in a difference map and freely refined with an isotropic displacement parameter. Other H atoms were placed in calculated positions and included in the final refinement in the riding model approximation, with displacement parameters derived from the parent atoms to which they are bonded. In the absence of significant anomalous dispersion effects, 1571 measured Friedel pairs were merged and the absolute configuration was arbitrarily assigned.

### Crystal data

**Table:**

C_42_H_54_O_6_	*Z* = 4
*M**_r_* = 654.85	*F*_000_ = 1416
Tetragonal, *P*4_1_2_1_2	*D*_x_ = 1.256 Mg m^−^^3^
Hall symbol: P 4abw 2nw	Mo *K*α radiation λ = 0.71073 Å
*a* = 13.5981 (10) Å	Cell parameters from 4034 reflections
*b* = 13.5981 (10) Å	θ = 4.8–54.2º
*c* = 18.7260 (19) Å	µ = 0.08 mm^−^^1^
α = 90º	*T* = 293 (2) K
β = 90º	Prismatic, colourless
γ = 90º	0.39 × 0.24 × 0.14 mm
*V* = 3462.6 (5) Å^3^	

### Data collection

**Table:**

Bruker APEX CCD area-detector diffractometer	2219 independent reflections
Radiation source: fine-focus sealed tube	1644 reflections with *I* > 2σ(*I*)
Monochromator: graphite	*R*_int_ = 0.110
*T* = 293(2) K	θ_max_ = 27.0º
φ and ω scans	θ_min_ = 1.9º
Absorption correction: multi-scan(SADABS; Bruker, 2000)	*h* = −16→17
*T*_min_ = 0.743, *T*_max_ = 0.990	*k* = −17→17
20532 measured reflections	*l* = −11→23

### Refinement

**Table:**

Refinement on *F*^2^	Secondary atom site location: difference Fourier map
Least-squares matrix: full	Hydrogen site location: inferred from neighbouring sites
*R*[*F*^2^ > 2σ(*F*^2^)] = 0.045	H atoms treated by a mixture of independent and constrained refinement
*wR*(*F*^2^) = 0.113	*w* = 1/[σ^2^(*F*_o_^2^) + (0.07*P*)^2^] where *P* = (*F*_o_^2^ + 2*F*_c_^2^)/3
*S* = 0.95	(Δ/σ)_max_ = 0.007
2219 reflections	Δρ_max_ = 0.23 e Å^−^^3^
225 parameters	Δρ_min_ = −0.20 e Å^−^^3^
Primary atom site location: structure-invariant direct methods	Extinction correction: none

### Fractional atomic coordinates and isotropic or equivalent isotropic displacement parameters (Å^2^)

**Table:**

	*x*	*y*	*z*	*U*_iso_*/*U*_eq_	
O1	0.15743 (13)	0.55657 (12)	0.08619 (8)	0.0429 (5)	
O2	0.12262 (16)	0.48461 (14)	0.33312 (11)	0.0530 (5)	
O3	0.34879 (12)	0.77308 (13)	0.13171 (8)	0.0415 (4)	
C1	0.1684 (2)	0.87117 (19)	−0.00763 (14)	0.0499 (7)	
H1A	0.1694	0.9034	0.0386	0.060*	
H1B	0.2340	0.8757	−0.0277	0.060*	
C2	0.0968 (3)	0.9254 (2)	−0.05633 (14)	0.0637 (9)	
H2A	0.0322	0.9261	−0.0345	0.076*	
H2B	0.1183	0.9930	−0.0619	0.076*	
C3	0.0904 (3)	0.8771 (2)	−0.12908 (15)	0.0681 (9)	
H3A	0.1538	0.8820	−0.1525	0.082*	
H3B	0.0430	0.9126	−0.1580	0.082*	
C4	0.0601 (3)	0.7683 (2)	−0.12559 (14)	0.0573 (8)	
C5	0.1302 (2)	0.71542 (19)	−0.07258 (12)	0.0431 (6)	
H5	0.1954	0.7217	−0.0944	0.052*	
C6	0.1141 (2)	0.6044 (2)	−0.06599 (13)	0.0474 (7)	
H6A	0.0577	0.5915	−0.0356	0.057*	
H6B	0.1010	0.5765	−0.1127	0.057*	
C7	0.2055 (2)	0.5575 (2)	−0.03409 (13)	0.0484 (7)	
H7A	0.1950	0.4871	−0.0303	0.058*	
H7B	0.2604	0.5679	−0.0664	0.058*	
C8	0.2325 (2)	0.59716 (19)	0.03840 (13)	0.0411 (6)	
C9	0.23190 (19)	0.71109 (17)	0.04069 (12)	0.0371 (6)	
H9	0.2905	0.7323	0.0145	0.045*	
C10	0.14213 (18)	0.76213 (18)	0.00287 (13)	0.0389 (6)	
C11	0.0747 (3)	0.7242 (3)	−0.20075 (15)	0.0850 (12)	
H11A	0.0422	0.7649	−0.2354	0.127*	
H11B	0.0472	0.6592	−0.2022	0.127*	
H11C	0.1436	0.7211	−0.2115	0.127*	
C12	−0.0502 (2)	0.7571 (3)	−0.10853 (18)	0.0746 (10)	
H12A	−0.0662	0.7956	−0.0672	0.112*	
H12B	−0.0647	0.6892	−0.0993	0.112*	
H12C	−0.0884	0.7795	−0.1485	0.112*	
C13	0.0492 (2)	0.7539 (2)	0.04922 (13)	0.0462 (6)	
H13A	0.0646	0.7722	0.0975	0.069*	
H13B	0.0257	0.6873	0.0483	0.069*	
H13C	−0.0006	0.7969	0.0308	0.069*	
C14	0.24566 (18)	0.74743 (19)	0.11748 (12)	0.0365 (5)	
H14	0.2065	0.8074	0.1231	0.044*	
C15	0.3325 (2)	0.5555 (2)	0.06039 (15)	0.0539 (7)	
H15A	0.3455	0.5721	0.1093	0.081*	
H15B	0.3829	0.5829	0.0305	0.081*	
H15C	0.3319	0.4852	0.0551	0.081*	
C16	0.21009 (17)	0.67425 (18)	0.17229 (12)	0.0355 (6)	
C17	0.22961 (17)	0.69613 (18)	0.24324 (13)	0.0351 (5)	
C18	0.20089 (17)	0.63566 (18)	0.29797 (12)	0.0359 (5)	
C19	0.15135 (18)	0.54863 (18)	0.28072 (13)	0.0384 (6)	
C20	0.13528 (19)	0.52376 (18)	0.21062 (13)	0.0401 (6)	
H20	0.1029	0.4655	0.1997	0.048*	
C21	0.16728 (18)	0.58544 (18)	0.15546 (13)	0.0367 (6)	
H2	0.149 (3)	0.513 (3)	0.3750 (19)	0.086 (11)*	

### Atomic displacement parameters (Å^2^)

**Table:**

	*U*^11^	*U*^22^	*U*^33^	*U*^12^	*U*^13^	*U*^23^
O1	0.0538 (11)	0.0380 (9)	0.0370 (9)	−0.0081 (8)	0.0003 (8)	−0.0055 (8)
O2	0.0695 (13)	0.0420 (10)	0.0474 (11)	−0.0153 (10)	0.0097 (10)	0.0052 (9)
O3	0.0395 (9)	0.0490 (10)	0.0360 (9)	−0.0103 (8)	0.0026 (8)	−0.0008 (8)
C1	0.0642 (18)	0.0418 (14)	0.0436 (14)	−0.0107 (13)	−0.0004 (14)	0.0011 (13)
C2	0.087 (2)	0.0442 (16)	0.0597 (19)	−0.0007 (17)	−0.0084 (18)	0.0115 (14)
C3	0.092 (3)	0.0619 (19)	0.0507 (18)	−0.0038 (18)	−0.0139 (18)	0.0132 (16)
C4	0.072 (2)	0.0574 (18)	0.0421 (15)	−0.0089 (15)	−0.0117 (15)	0.0084 (15)
C5	0.0527 (15)	0.0435 (14)	0.0333 (13)	−0.0078 (13)	0.0020 (12)	−0.0012 (11)
C6	0.0624 (18)	0.0469 (15)	0.0329 (13)	−0.0116 (14)	−0.0030 (13)	−0.0100 (12)
C7	0.0604 (18)	0.0436 (15)	0.0412 (14)	0.0014 (13)	0.0053 (14)	−0.0076 (12)
C8	0.0461 (15)	0.0397 (13)	0.0373 (13)	0.0023 (12)	0.0052 (12)	−0.0056 (12)
C9	0.0410 (13)	0.0376 (12)	0.0328 (13)	−0.0041 (11)	0.0052 (11)	−0.0029 (11)
C10	0.0441 (14)	0.0387 (13)	0.0339 (12)	−0.0051 (11)	0.0019 (11)	0.0006 (11)
C11	0.134 (4)	0.081 (2)	0.0400 (18)	−0.012 (3)	−0.016 (2)	0.0019 (17)
C12	0.071 (2)	0.080 (2)	0.073 (2)	−0.0043 (19)	−0.0276 (18)	0.006 (2)
C13	0.0454 (15)	0.0527 (16)	0.0405 (14)	0.0016 (14)	0.0047 (12)	−0.0006 (13)
C14	0.0366 (12)	0.0396 (13)	0.0332 (13)	−0.0056 (11)	−0.0004 (10)	0.0001 (11)
C15	0.0551 (18)	0.0540 (17)	0.0526 (16)	0.0122 (14)	0.0044 (14)	−0.0041 (14)
C16	0.0325 (12)	0.0365 (13)	0.0375 (13)	−0.0026 (10)	0.0022 (11)	−0.0009 (11)
C17	0.0318 (12)	0.0374 (12)	0.0360 (13)	−0.0021 (10)	0.0019 (11)	−0.0025 (11)
C18	0.0353 (12)	0.0404 (13)	0.0321 (13)	−0.0011 (11)	0.0022 (10)	−0.0017 (11)
C19	0.0402 (14)	0.0343 (13)	0.0407 (13)	−0.0035 (11)	0.0071 (11)	0.0036 (11)
C20	0.0432 (14)	0.0341 (12)	0.0430 (14)	−0.0062 (11)	0.0003 (12)	−0.0051 (11)
C21	0.0380 (13)	0.0375 (13)	0.0345 (13)	−0.0014 (11)	0.0002 (11)	−0.0047 (11)

### Geometric parameters (Å, °)

**Table:**

O1—C21	1.362 (3)	C8—C9	1.550 (3)
O1—C8	1.465 (3)	C9—C14	1.532 (3)
O2—C19	1.369 (3)	C9—C10	1.573 (3)
O2—H2	0.95 (4)	C9—H9	0.9800
O3—C18^i^	1.380 (3)	C10—C13	1.537 (3)
O3—C14	1.469 (3)	C11—H11A	0.9600
C1—C2	1.524 (4)	C11—H11B	0.9600
C1—C10	1.538 (3)	C11—H11C	0.9600
C1—H1A	0.9700	C12—H12A	0.9600
C1—H1B	0.9700	C12—H12B	0.9600
C2—C3	1.515 (4)	C12—H12C	0.9600
C2—H2A	0.9700	C13—H13A	0.9600
C2—H2B	0.9700	C13—H13B	0.9600
C3—C4	1.536 (5)	C13—H13C	0.9600
C3—H3A	0.9700	C14—C16	1.509 (3)
C3—H3B	0.9700	C14—H14	0.9800
C4—C12	1.541 (5)	C15—H15A	0.9600
C4—C11	1.543 (4)	C15—H15B	0.9600
C4—C5	1.553 (4)	C15—H15C	0.9600
C5—C6	1.530 (4)	C16—C21	1.377 (3)
C5—C10	1.558 (3)	C16—C17	1.387 (3)
C5—H5	0.9800	C17—C18	1.371 (3)
C6—C7	1.520 (4)	C17—C17^i^	1.450 (5)
C6—H6A	0.9700	C18—O3^i^	1.380 (3)
C6—H6B	0.9700	C18—C19	1.399 (4)
C7—C8	1.506 (3)	C19—C20	1.373 (3)
C7—H7A	0.9700	C20—C21	1.400 (3)
C7—H7B	0.9700	C20—H20	0.9300
C8—C15	1.530 (4)		
			
C21—O1—C8	113.87 (18)	C10—C9—H9	106.0
C19—O2—H2	103 (2)	C13—C10—C1	109.5 (2)
C18^i^—O3—C14	112.36 (18)	C13—C10—C5	113.4 (2)
C2—C1—C10	113.2 (2)	C1—C10—C5	107.5 (2)
C2—C1—H1A	108.9	C13—C10—C9	110.58 (19)
C10—C1—H1A	108.9	C1—C10—C9	107.6 (2)
C2—C1—H1B	108.9	C5—C10—C9	108.0 (2)
C10—C1—H1B	108.9	C4—C11—H11A	109.5
H1A—C1—H1B	107.7	C4—C11—H11B	109.5
C3—C2—C1	111.4 (3)	H11A—C11—H11B	109.5
C3—C2—H2A	109.3	C4—C11—H11C	109.5
C1—C2—H2A	109.3	H11A—C11—H11C	109.5
C3—C2—H2B	109.3	H11B—C11—H11C	109.5
C1—C2—H2B	109.3	C4—C12—H12A	109.5
H2A—C2—H2B	108.0	C4—C12—H12B	109.5
C2—C3—C4	113.2 (3)	H12A—C12—H12B	109.5
C2—C3—H3A	108.9	C4—C12—H12C	109.5
C4—C3—H3A	108.9	H12A—C12—H12C	109.5
C2—C3—H3B	108.9	H12B—C12—H12C	109.5
C4—C3—H3B	108.9	C10—C13—H13A	109.5
H3A—C3—H3B	107.7	C10—C13—H13B	109.5
C3—C4—C12	111.4 (3)	H13A—C13—H13B	109.5
C3—C4—C11	107.5 (3)	C10—C13—H13C	109.5
C12—C4—C11	106.0 (3)	H13A—C13—H13C	109.5
C3—C4—C5	108.0 (2)	H13B—C13—H13C	109.5
C12—C4—C5	114.8 (2)	O3—C14—C16	109.82 (18)
C11—C4—C5	108.9 (3)	O3—C14—C9	111.31 (19)
C6—C5—C4	114.9 (2)	C16—C14—C9	112.7 (2)
C6—C5—C10	110.1 (2)	O3—C14—H14	107.6
C4—C5—C10	117.1 (2)	C16—C14—H14	107.6
C6—C5—H5	104.4	C9—C14—H14	107.6
C4—C5—H5	104.4	C8—C15—H15A	109.5
C10—C5—H5	104.4	C8—C15—H15B	109.5
C7—C6—C5	109.2 (2)	H15A—C15—H15B	109.5
C7—C6—H6A	109.8	C8—C15—H15C	109.5
C5—C6—H6A	109.8	H15A—C15—H15C	109.5
C7—C6—H6B	109.8	H15B—C15—H15C	109.5
C5—C6—H6B	109.8	C21—C16—C17	119.2 (2)
H6A—C6—H6B	108.3	C21—C16—C14	123.9 (2)
C8—C7—C6	113.7 (2)	C17—C16—C14	116.7 (2)
C8—C7—H7A	108.8	C18—C17—C16	122.2 (2)
C6—C7—H7A	108.8	C18—C17—C17^i^	119.0 (3)
C8—C7—H7B	108.8	C16—C17—C17^i^	116.7 (3)
C6—C7—H7B	108.8	C17—C18—O3^i^	123.3 (2)
H7A—C7—H7B	107.7	C17—C18—C19	118.1 (2)
O1—C8—C7	104.3 (2)	O3^i^—C18—C19	118.3 (2)
O1—C8—C15	108.4 (2)	O2—C19—C20	118.9 (2)
C7—C8—C15	109.0 (2)	O2—C19—C18	120.6 (2)
O1—C8—C9	110.86 (19)	C20—C19—C18	120.4 (2)
C7—C8—C9	112.5 (2)	C19—C20—C21	120.6 (2)
C15—C8—C9	111.6 (2)	C19—C20—H20	119.7
C14—C9—C8	110.36 (19)	C21—C20—H20	119.7
C14—C9—C10	112.0 (2)	O1—C21—C16	120.8 (2)
C8—C9—C10	115.6 (2)	O1—C21—C20	120.0 (2)
C14—C9—H9	106.0	C16—C21—C20	119.2 (2)
C8—C9—H9	106.0		
			
C10—C1—C2—C3	−57.4 (3)	C14—C9—C10—C1	67.9 (2)
C1—C2—C3—C4	57.2 (4)	C8—C9—C10—C1	−164.5 (2)
C2—C3—C4—C12	74.3 (3)	C14—C9—C10—C5	−176.30 (19)
C2—C3—C4—C11	−169.9 (3)	C8—C9—C10—C5	−48.7 (3)
C2—C3—C4—C5	−52.6 (4)	C18^i^—O3—C14—C16	−50.0 (2)
C3—C4—C5—C6	−176.6 (3)	C18^i^—O3—C14—C9	−175.53 (19)
C12—C4—C5—C6	58.5 (4)	C8—C9—C14—O3	97.3 (2)
C11—C4—C5—C6	−60.1 (3)	C10—C9—C14—O3	−132.3 (2)
C3—C4—C5—C10	52.0 (3)	C8—C9—C14—C16	−26.6 (3)
C12—C4—C5—C10	−73.0 (3)	C10—C9—C14—C16	103.8 (2)
C11—C4—C5—C10	168.4 (2)	O3—C14—C16—C21	−126.5 (2)
C4—C5—C6—C7	160.9 (2)	C9—C14—C16—C21	−1.7 (3)
C10—C5—C6—C7	−64.4 (3)	O3—C14—C16—C17	48.3 (3)
C5—C6—C7—C8	58.8 (3)	C9—C14—C16—C17	173.0 (2)
C21—O1—C8—C7	−178.8 (2)	C21—C16—C17—C18	−5.2 (4)
C21—O1—C8—C15	65.2 (3)	C14—C16—C17—C18	179.8 (2)
C21—O1—C8—C9	−57.6 (3)	C21—C16—C17—C17^i^	158.50 (17)
C6—C7—C8—O1	72.2 (3)	C14—C16—C17—C17^i^	−16.5 (2)
C6—C7—C8—C15	−172.2 (2)	C16—C17—C18—O3^i^	174.0 (2)
C6—C7—C8—C9	−47.9 (3)	C17^i^—C17—C18—O3^i^	10.7 (3)
O1—C8—C9—C14	55.9 (3)	C16—C17—C18—C19	0.8 (4)
C7—C8—C9—C14	172.2 (2)	C17^i^—C17—C18—C19	−162.49 (17)
C15—C8—C9—C14	−64.9 (3)	C17—C18—C19—O2	178.8 (2)
O1—C8—C9—C10	−72.5 (3)	O3^i^—C18—C19—O2	5.2 (3)
C7—C8—C9—C10	43.8 (3)	C17—C18—C19—C20	2.1 (4)
C15—C8—C9—C10	166.6 (2)	O3^i^—C18—C19—C20	−171.5 (2)
C2—C1—C10—C13	−70.9 (3)	O2—C19—C20—C21	−177.3 (2)
C2—C1—C10—C5	52.7 (3)	C18—C19—C20—C21	−0.6 (4)
C2—C1—C10—C9	168.9 (2)	C8—O1—C21—C16	28.2 (3)
C6—C5—C10—C13	−64.4 (3)	C8—O1—C21—C20	−150.7 (2)
C4—C5—C10—C13	69.2 (3)	C17—C16—C21—O1	−172.3 (2)
C6—C5—C10—C1	174.4 (2)	C14—C16—C21—O1	2.3 (4)
C4—C5—C10—C1	−52.0 (3)	C17—C16—C21—C20	6.5 (4)
C6—C5—C10—C9	58.5 (3)	C14—C16—C21—C20	−178.9 (2)
C4—C5—C10—C9	−167.9 (2)	C19—C20—C21—O1	175.1 (2)
C14—C9—C10—C13	−51.7 (3)	C19—C20—C21—C16	−3.7 (4)
C8—C9—C10—C13	75.9 (3)		

Symmetry codes: (i) −*y*+1, −*x*+1, −*z*+1/2.

### Hydrogen-bond geometry (Å, °)

**Table:**

*D*—H···*A*	*D*—H	H···*A*	*D*···*A*	*D*—H···*A*
O2—H2···O3^i^	0.95 (4)	2.16 (4)	2.753 (3)	120 (3)

Symmetry codes: (i) −*y*+1, −*x*+1, −*z*+1/2.

## References

[bb1] Alvarez-Manzaneda, E. J., Chahboun, R., Barranco Pérez, I., Cabrera, E., Alvarez, E. & Alvarez-Manzaneda, R. (2005). *Org. Lett.* **7**, 1477–1480. 10.1021/ol047332j 15816731

[bb2] Alvarez-Manzaneda, E. J., Chahboun, R., Cabrera, E., Alvarez, E., Haidour, A., Ramos, J. M., Alvarez-Manzaneda, R., Hmamouchi, M. & Bouanou, H. (2007). *J. Org. Chem.* **72**, 3332–3339. 10.1021/jo0626663 17388632

[bb3] Amade, P., Chevelot, L., Perzanowski, H. P. & Scheuer, P. J. (1983). *Helv. Chim. Acta*, **66**, 1672–1675.

[bb4] Barrero, A. F., Alvarez-Manzaneda, E. J., Herrador, M. M., Valdivia, M. V. & Chahboun, R. (1998). *Tetrahedron*, **39**, 2425–2428.

[bb5] Barrero, A. F., Alvarez-Miranda, E. J., Chahboun, R., Cortés, M. & Armstrong, V. (1999). *Tetrahedron*, **55**, 15181–15208.

[bb6] Bruker (2000). *SMART*, *SAINT* and *SADABS* Bruker AXS Inc., Madison, Wisconsin, USA.

[bb7] Castro, M. E., González-Iriarte, M., Barrero, A. F., Salvador-Tormo, N., Muñoz-Chápuli, R., Medina, M. A. & Quesada, A. R. (2004). *Int. J. Cancer*, **20**, 31–38. 10.1002/ijc.20068 15054866

[bb8] Ciavatta, M. L., Lopez Gresa, M. P., Gavagnin, M., Romero, V., Melck, D., Manzo, E., Guo, Y.-W., van Soest, R. & Cimino, G. (2007). *Tetrahedron*, **63**, 1380–1384.

[bb9] Hamann, M. T. (2003). *Curr. Pharm. Des.* **9**, 879–889. 10.2174/1381612033455297 12678872

[bb10] Kohmoto, S., McConnell, O. J., Wright, A., Koehn, F., Thompson, W., Lui, M. & Snader, K. M. (1987). *J. Nat. Prod.* **50**, 336–336. 10.1021/np50050a064 3655802

[bb11] Longley, R. E., McConnell, O. J., Essich, E. & Harmody, D. (1993). *J. Nat. Prod.* **56**, 915–920. 10.1021/np50096a015 8350092

[bb12] Sheldrick, G. M. (2008). *Acta Cryst.* A**64**, 112–122. 10.1107/S0108767307043930 18156677

[bb13] Takamatsu, S., Hodges, T. W., Rajbhandari, I., Gerwick, W. H., Hamann, M. T. & Nagle, D. G. (2003). *J. Nat. Prod.* **66**, 605–608. 10.1021/np0204038 PMC4969048 12762791

